# The Impact of Social Media on Medical Professionalism: A Systematic Qualitative Review of Challenges and Opportunities

**DOI:** 10.2196/jmir.2708

**Published:** 2013-08-28

**Authors:** Fatemeh Gholami-Kordkheili, Verina Wild, Daniel Strech

**Affiliations:** ^1^Institute for History, Ethics and Philosophy of MedicineCELLS-Centre for Ethics and Law in the Life ScienceHannover Medical SchoolHannoverGermany; ^2^Institute for Biomedical EthicsUniversity of ZurichZurichSwitzerland

**Keywords:** social media, professionalism, facebook, blogs, Twitter, health policy

## Abstract

**Background:**

The rising impact of social media on the private and working lives of health care professionals has made researchers and health care institutions study and rethink the concept and content of medical professionalism in the digital age. In the last decade, several specific policies, original research studies, and comments have been published on the responsible use of social media by health care professionals. However, there is no systematic literature review that analyzes the full spectrum of (1) social media–related challenges imposed on medical professionalism and (2) social media–related opportunities to both undermine and improve medical professionalism.

**Objective:**

The aim of this systematic qualitative review is to present this full spectrum of social media–related challenges and opportunities.

**Methods:**

We performed a systematic literature search in PubMed (restricted to English and German literature published between 2002 and 2011) for papers that address social media–related challenges and opportunities for medical professionalism. To operationalize “medical professionalism”, we refer to the 10 commitments presented in the physicians’ charter “Medical professionalism in the new millennium” published by the ABIM Foundation. We applied qualitative text analysis to categorize the spectrum of social media–related challenges and opportunities for medical professionalism.

**Results:**

The literature review retrieved 108 references, consisting of 46 original research studies and 62 commentaries, editorials, or opinion papers. All references together mentioned a spectrum of 23 broad and 12 further-specified, narrow categories for social media–related opportunities (n=10) and challenges (n=13) for medical professionalism, grouped under the 10 commitments of the physicians’ charter.

**Conclusions:**

The accommodation of the traditional core values of medicine to the characteristics of social media presents opportunities as well as challenges for medical professionalism. As a profession that is entitled to self-regulation, health care professionals should proactively approach these challenges and seize the opportunities. There should be room to foster interprofessional and intergenerational dialogue (and eventually guidelines and policies) on both challenges and opportunities of social media in modern health care. This review builds a unique source of information that can inform further research and policy development in this regard.

## Introduction

Professionalism is the basis of medicine’s contract with society [1]

In 2002, the European Federation of Internal Medicine, the American College of Physicians-American Society of Internal Medicine (ACP-ASIM), and the American Board of Internal Medicine (ABIM) felt it necessary to renew the sense of professionalism due to changing market forces. The result of these efforts was a new physicians’ charter, which claimed to apply to physicians throughout the world.

Ten years later, the rising influence of social media in our private and professional lives is a new force that affects our understanding of medical professionalism. Social media, as a part of the Web 2.0, include blogs, wikis, podcasts, and social networking platforms such as Twitter, LinkedIn, YouTube, and Facebook, to name just a few. In contrast to websites where people are limited to the passive viewing of content, Web 2.0 tools are people-based knowledge sharing, learning, social interaction, and collective intelligence tools that support knowledge collaboration, exchange, sharing, and creation [2]. Thompson et al reported in 2008 that 45% of medical trainees, 64% of medical students, and 13% of medical residents had Facebook accounts [3].

The asymmetry of disclosure in the doctor-patient relationship was emphasized long before social media [4]. Today, social media allow patients to gather increasingly more information about their doctors’ private and professional life. Excessive self-disclosure from the side of the physician is generally regarded as a boundary violation in the patient-physician treatment relationship [5]. Disclosure of this kind of personal information on a social networking site is usually not aimed at patients, but patients might nevertheless access this information [6].

Persistence, searchability, replicability, and invisible audiences are unique characteristics of Facebook and other social media platforms [7], which form—based on the ease of searching and storing digital information—a “permanent” digital fingerprint and online reputation. Once information is online, it is extremely difficult to remove it (if at all) and it can quickly spread beyond one’s control. A moment of rashness could have unintended and irreversible consequences in the future such as suspension from medical school, loss of employment as a physician, and loss of trust in the medical profession [8]. It could concern future or current employment candidacy, or current employment and training conditions. There are already cases of students, trainees, or medical staff being dismissed because of their “unprofessional” online image [9,10].

However, the reduction of power imbalances between patients and doctors has been shown to improve patient confidence in starting, stopping, or making changes to treatment regimens [11]. Social media may also help to distribute precise health information to a larger group of individuals than ever before. But is online available medical information reliable? Who provides the medical information on blogs, YouTube, Twitter, and Facebook? In 2008, there were 1434 medical-related blogs; however, only 279 were actually written by medical professionals [12]. As advertising and business interests strongly influence the order of search engine listings [13], it might be advisable for the medical and dental professions to proactively refer patients to high-quality sources of medical online information [14,15].

Universities and medical organizations, especially in the United States (such as the American Medical Association, AMA) and United Kingdom, have started to develop guidelines and policies for health care professionals concerning proper social media use. In order to foster awareness, courses on handling social media associated with medical professionalism have been implemented in the professional curricula [16]. The recently published position paper on online medical professionalism by the American College of Physicians and the Federation of State Medical Boards provides the latest recommendations on strategies for physician-physician communication that aims at preserving confidentiality while best profiting from the new technologies of social media [17].

The importance of social media is also indicated by the increasing number of scientific publications that deal with them in the medical context. While our search (see Methods) found a total of 1471 publications focusing on social media on PubMed in December 2011, by the end of December 2012 there were 2330 hits.

To our knowledge, there is no systematic literature review that analyses the full spectrum of (1) social media–related challenges to medical professionalism and (2) social media–related opportunities to either undermine or improve medical professionalism. The aim of this systematic qualitative review is to present this spectrum.

## Methods

### Literature Search and Eligibility Criteria

**Figure 1:**
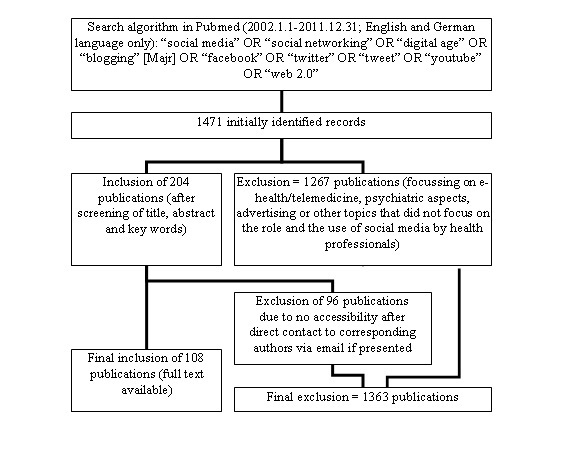
Flowchart illustrating identified references.

In December 2011, we searched PubMed with the following terms: “social media” OR “social networking” OR “digital age” OR “blogging” [Majr] OR “facebook” OR “twitter” OR “tweet” OR “youtube” OR “Web 2.0”. The search was restricted to English or German language papers. Publications before 2002 were excluded because all major social media platforms were founded after 2002: MySpace was founded in 2003 [18], Facebook in 2004 [19], and Twitter in 2006 [20]. We included publications focusing on the use of social media by health professionals, challenges imposed on health professionals by social media use, and ethical considerations concerning the relationship between patients and health professionals in the Internet era. We excluded publications focusing on eHealth/telemedicine, addiction, and other psychiatric issues related to social media, and advertising or marketing. See Figure 1.

### Extraction and Categorization of Social Media–Related Opportunities and Challenges for Medical Professionalism

Our aim was to develop a qualitative framework of narrow and broad categories of social media–related opportunities and challenges for medical professionalism that best accommodated the opportunities and challenges mentioned in the included publications.

To operationalize “medical professionalism”, we referred to the 10 commitments/professional responsibilities presented in the physicians’ charter, “Medical professionalism in the new millennium” published by the ABIM Foundation, the ACP-ASIM Foundation, and the European Federation of Internal Medicine. To our knowledge, the physicians’ charter is the most widely accepted and most often cited framework for medical professionalism. It has been endorsed by over 90 professional societies worldwide. Since its publication in 2002 in several journals, it has been cited more than 900 times (as assessed by Scopus). The 10 commitments are (1) professional competence, (2) honesty with patients, (3) patient confidentiality, (4) maintaining appropriate relations with patients, (5) improving quality of care, (6) improving access to care, (7) a just distribution of finite resources, (8) scientific knowledge, (9) maintaining trust by managing conflicts of interest, and (10) professional responsibilities.

We employed the 10 commitments of medical professionalism as our matrix to guide the identification of text passages that mention social media–related opportunities or challenges for medical professionalism. Mentions of such opportunities and challenges in different papers were compared. Broad and narrow categories were developed for similar mentions of opportunities and challenges. According to our matrix, these broad and narrow categories were grouped under the 10 commitments.

To ensure the validity of coding as well as intercoder reliability, we employed the following procedure: 3 authors (FG, VW, DS) identified and initially categorized opportunities and challenges (based on the above described extraction matrix) independently in a subsample of 5 publications. The authors discussed whether paragraphs mentioned opportunities and challenges and how they should be categorized. The remaining 103 publications were grouped in three clusters of 60, 23, and 20 publications. One author (FG) with an MD degree then extracted and categorized social media-related opportunities and challenges from this first cluster of publications. The result was a first version of the spectrum of social media-related opportunities and challenges grouped under the 10 commitments. The second and third clusters of references were then used to check theoretical saturation of the spectrum. Theoretical saturation means that no new categories can be generated [21]. Once theoretical saturation was reached for broad categories, the other authors (DS, VW), with professional backgrounds in bioethics, clinical psychiatry, internal medicine, philosophy, and health services research, checked the extraction and categorization of opportunities and challenges in a random sample of 25 publications. Coding problems were resolved by frequent meetings and discussions between all authors.

## Results

From 1471 initial hits in PubMed, we finally included 108 in this review. The 108 references consist of 46 original research studies and 62 commentaries, editorials, and opinion papers. The majority are from the United States (79 publications), followed by 15 from the United Kingdom. Other papers come from Canada (5 publications), Ireland (3 publications), Australia (2 publications), and Germany, Peru, France, and New Zealand (1 publication each). The sample consists of one article published in 2006, three in 2008, 13 in 2009, 21 in 2010, and 70 articles in 2011.

We identified 23 broad and 12 further-specified narrow categories for social media–related opportunities (n=10) and challenges (n=13) for medical professionalism, grouped under the 10 commitments of the physicians’ charter.

For example, for the first commitment “professional competence”, we identified four broad categories for opportunities (A-D) and one broad category for a challenge (E): (A) Employing social media as a tool for improved information sharing, (B) Increasing the involvement by doctors in under-served areas, (C) Committing to life-long learning supported by the use of social media, (D) Mentoring student’s reasonable engagement in social media, and (E) Ensuring evidence-based Continuing Medical Education in the environment of social media. Some of these broad categories are specialized into more narrow categories. For example, the broad category (A) Employing Social Media as a tool for improved information sharing was specified into five narrow categories: (A1) Fast and boundless dissemination of news and experience, (A2) Collaboration on challenging cases, (A3) Improving access to and benefits of conferences and news exchange, (A4) Sharing information on physician-only social media sites, and (A5) Accessing news/information from professional organizations. One of many original text passages extracted from the narrow category (A1) is “With Internet-based tools, physicians are no longer limited by geography, specialty, and time zone in their attempts to connect, engage, and learn from each other” [22]. For technical reasons and for didactic purposes, we restrict our presentation to one exemplary text passage for each of the 33 narrow categories (see Multimedia Appendix 1 for these findings; [4,9,14,22-41]).

## Discussion

### Principal Findings

This systematic qualitative review presents the full spectrum of social media–related opportunities and challenges for medical professionalism as they are currently discussed in original research studies, commentaries, editorials, or opinion papers published in scientific journals listed in PubMed. Thereby it builds a unique source of knowledge that can inform further research and policy development in the intersection of social media and medical professionalism.

The need for policies on the use of social media by medical professionals, trainees, and students has already been addressed by some universities [42] and also by institutions such as the AMA [43]. The AMA policy “Medical professionalism in the digital age”, which was adopted in November 2010, presents general recommendations. It encourages the medical practitioner to “weigh a number of considerations” when it comes to social media. The gist of the policy is to preserve patient privacy and confidentiality in all environments, to avoid excessive self-disclosure by using adequate privacy settings, being aware that they are not absolute, and routinely monitoring one’s online presence. It stresses the necessity of maintaining appropriate patient/physician boundaries, and in doing so to consider the separation of professional and personal online content. The policy tries to raise awareness of the professional’s responsibility to bring posted unprofessional content to the attention of the individual in question or to inform appropriate authorities, as those failures may affect the medical professional’s reputation among patients and colleagues and may undermine public trust. Even though the above-mentioned issues (which almost all describe challenges) are important, the AMA policy neither illustrates a more differentiated view of social media-related challenges, nor does it acknowledge social media-related opportunities and the need to address them appropriately. Such opportunities include, for instance, improvement in sharing information, access to care, and quality of care, etc [43] (see Multimedia Appendix 1).

The University of Florida, for example, recognizes the relevance of social media as a current form of communication. However, it also focuses on challenges and distinguishes “strictly forbidden” from “strongly discouraged” online interactions, which could be the basis for disciplinary actions. Violating patient confidentiality, reporting private academic information, and neglecting official work commitments when interacting online are strictly forbidden actions. Strongly discouraged actions include use of vulgar language, implying disrespect for any individual due to age, race, gender, etc, presentation of alcohol misuse, substance abuse, sexual promiscuity, and posting unflattering material on another individual’s website. The policy tries to raise awareness that a mature, responsible, and professional attitude should also be displayed when interacting online privately and to think twice before posting any material because online privacy measures might be unreliable [44].

Although it is a laudable first step that both the AMA policy and the University of Florida policy explicitly address some social media–related challenges for medical professionalism, in their current version they address neither the full spectrum of challenges nor any of the social media–related opportunities (see Multimedia Appendix 1). In general, social media–related challenges are more frequently discussed in the reviewed publications than social media–related opportunities. But as the relevance of social media might further increase, there is an ongoing demand for a critical and constructive discussion about, and guidelines/recommendations on, how to best possibly address the multifaceted spectrum of challenges and opportunities.

Particularly among medical students and young professionals on the one hand and educators and practicing physicians on the other, there may be a different attitude towards the use of social media. Prensky introduced the distinction of digital natives and digital immigrants that is often referred to in today’s debate on online medical professionalism [23,45]. Current trainees and medical students born after 1980 are considered as digital natives, as they grew up in a world where using technology (eg, computers, the Internet, text messaging, blogging, and SMS text messaging) was already integrated within their education, patterns of establishing/maintaining relationships, and means of self-expression. Older faculty who completed their training before 1980 are considered digital immigrants because a good number of them experience a challenge to continually adopt to the particularities of the digital age with which their students are likely more familiar [23]. However, a sharp distinction between digital natives and digital immigrants might blur in the near future, and further distinctions across digital natives might occur. We have, for example, anecdotal evidence that some current medical students do not understand how to use email for personal communication due to unfamiliarity; instead they try to use it as if it were Facebook or Twitter.

In addition, professionalism is acquired over time and is best learned within the practice community and specifically through observation of role models [46]. However, mentoring and observation of role models as a vital component of developing professionalism might face difficulties in the digital age, with different generations of physicians practicing in parallel [23]. This particular situation further favors policies that capture the broad spectrum of challenges and opportunities for medical professionalism with respect to social media.

### Limitations

There are some limitations to our review: we screened only contributions published (in different types of publications) in scientific journals listed in PubMed. Only German and English publications were considered. Only publications after 2002 were included, due to the fact that all major social media platforms were founded after the year 2002 [18-20]. While our search revealed 1471 references listed in PubMed for the years 2002-2011, another 982 references are listed in PubMed in 2012 that could not be included in this review. Because our review already included more than 100 references published in journals from various subspecialties and because we reached theoretical saturation for our broad categories of opportunities and challenges, we felt justified in limiting our review to the described literature search.

Because the findings of our review are purely descriptive and we did not provide additional normative analysis to each of the identified challenges and opportunities, we refrain from concluding on how these challenges and opportunities should be best addressed in medical practice. However, the recently published position paper by the American College of Physicians and the Federation of State Medical Board presents several distinguished implications of online activities for patients, physicians, and the medical profession and provides recommendations on how to avoid potential pitfalls while best using social media technologies [17]. Also, other in-depth analyses result in specific suggestions on how to deal with social media-related challenges and opportunities [16,47]. However, none of the above mentioned policy and recommendation papers refer to a systematicially and transparently derived account of challenges and opportunities.

### Conclusions

The integration of traditional core values of medicine (privacy, confidentiality, one-on-one interactions, and formal conduct) and the culture of social media (which tends to value sharing and openness, connection, transparency, and informality) present opportunities as well as challenges for medical professionalism [24]. As a profession that is entitled to self-regulation, health care professionals should proactively approach these challenges and make use of the opportunities. There should be room for fostering interprofessional and intergenerational dialogue (eg, digital natives/digital immigrants). There is a further demand for research and policy development to integrate the broad spectrum of social media’s opportunities and challenges into the current existing frameworks for medical professionalism. This review builds a unique source of information that can inform further research and policy development in this regard.

## Multimedia Appendix 1

The spectrum of social media–related opportunities and challenges for medical professionalism.

## Abbreviations

ABIM: American Board of Internal Medicine
ACP-ASIM: American College of Physicians-American Society of Internal Medicine
AMA: American Medical Association
