# Supplementary material for: The Impact of Social Media on Medical Professionalism: A Systematic Qualitative Review of Challenges and Opportunities
Source: J Med Internet Res. 2013 Aug 28;15(8):e184. doi: 10.2196/jmir.2708 (PMC3758042; doi:10.2196/jmir.2708)
Supplement: Supplementary file 1 [file jmir_v15i8e184_app1.pdf]

Multimedia Appendix 1. The spectrum of social media–related opportunities and challenges for medical professionalism (number of quotations are given in round parentheses and references are given in square brackets).

**1. Commitment to professional competence**	
1.1. Opportunity : Employing social media as a tool for improved information sharing	
1.1.1. Fast and boundless dissemination of news and experience (67)	With Internet-based tools, physicians are no longer limited by geography, specialty, and time zone in their attempts to connect, engage, and learn from each other.[22]
1.1.2. Collaboration on demanding cases (14)	Medicine, by nature, is a team sport, and often two heads are better than one. While physicians of yesteryear relied on “curbside” consults with colleagues working in close proximity, today’s physicians can rely on other physicians whom they have never met, but are connected to on social media to assist them in their clinical practice[31]
1.1.3. Improving access to and benefits of conferences and news exchange (8)	Most conferences I now attend seem to have an accompanying blog, where readers can go to learn more about the conference proceedings and presentations. Accompanying videos and photographs can provide a multimedia experience for the reader.[32]
1.1.4. Sharing information on physician-only social media sites (27)	Over the past few years, several physician-only online social networks have been launched, such as Medscape Connect (http://www.medscape.com/ connect) and Sermo (http://www.sermo.com/). These sites, which are free to join and use, allow physicians from around the world to connect, ask/answer questions, and collaborate on difficult cases with colleagues.[33]
1.1.5. Accessing news/ information from professional organizations (25)	Approximately 1,100 hospitals nationwide have established their presence on Facebook, Twitter, YouTube LinkedIn, or blogs. Information conveyed across these sites from hospitals range from breaking health news to ongoing organizational events.[34]
1.2. Opportunity: Increasing the involvement by doctors in under-served areas (5)	
	The benefits of online professional networking are many and are primarily centered around sharing information in a knowledge ecosystem. Additionally, these tools can level the playing field for doctors in rural or underserved areas by more rapidly and deeply disseminating modern-day techniques, thought processes, and knowledge-based insights.[22]
1.3. Opportunity: Committing to life-long learning supported by the use of social media	
1.3.1. Improvement in learning patterns regarding graduate students (16)	Wikis can act as a shared knowledge-base for students to collate their identified resources relating to professionalism in one, accessible place. Having this shared knowledge-base online allowed students to continue their learning in-between their face-to-face sessions.[35]
1.3.2. Facilitating continuing education for post-graduates/clinician (28)	The advantage of having Continuing Professional Development material available to complete online is of course the flexibility; practitioners can learn in their own time and are not restricted to set conferences and talks.[14]
1.4. Opportunity: Mentoring student’s reasonable engagement in social media (66)	
	Veterinary educators have an important role in helping veterinarians-in-training navigate the blurring line between private and professional identities brought on by social media.[36]
1.5. Challenge: Ensuring evidence-based Continuing Medical Education in the environment of Social Media (7)	
	There is the option to take online courses, which are recognized for part fulfilment of US CPD requirements. Membership is free and a browse of the site is recommended, but it should be noted that most of the information appears to be in the form of clinical opinion rather than evidence-based data, and caution is required![14]
**2. Commitment to honesty with patients**	
2.1. Challenge: Managing disclosure of additional patient information that a physician gathered online (21)	

	Once healthcare providers seek patient SMS information, they are then charged with how to best respond. Responses become even more critical if a patient posts information that could affect the patient's physical and/or emotional health.[37]
**3. Commitment to patient confidentiality**	
3.1. Opportunity: Using de-identified, respectful patient stories to encourage reflection, empathy, understanding (11)	
	For physicians, sharing patients' stories that are de-identified and respectful, on personal blogs or social networking sites, can encourage reflection, empathy, and understanding.[9]
3.2. Challenge: Maintaining patient privacy while engaging in social media (35)	
	Another concern with Facebook is the potential for breaching patient privacy in a non-clinical setting. A post about a challenging patient may contain enough recognizable information to compromise confidentiality.[4]
3.3. Challenge: Dealing with social media as a source for (third-party) misunderstanding and misinterpretation (16)	
	The intermingling of physicians with patients in public domains, online and otherwise, leaves a lot of room for speculation and misunderstanding. Online interactions are memorialized and subject to third-party scrutiny and misinterpretation.[22]
**4. Commitment to maintaining appropriate relations with patients**	
4.1. Challenge: Reasonable self-disclosure of health care providers in social media	
4.1.1. Deliberate self disclosure induced by the health care provider (52)	For digital natives, the idea that some of this information should remain private, or that it might impact the patient-doctor relationship, seems strange. They have grown up in a "hyperpersonal" world, in which it feels comfortable to digitally expose oneself online. [27]
4.1.2. Unintentional self-disclosure induced through other social media users (28)	The availability of search engines has truly altered the landscape of privacy, anonymity, and self-disclosure in the clinical setting. Psychiatrists must now assume that patients know a good deal about them and must re-think their stance that their private life is beyond reach of the patient.[4]
4.1.3. Solutions to avoid excessive self disclosure online (30)	One solution for the problems associated with using social media is to have personal and professional pages or to use privacy settings to limit what can be viewed.[38]
4.1.4. Dealing with the impact of excessive self disclosure on the professional medical reputation (33)	The results from our study suggest that PDs of surgical residency programs are beginning to embrace this emerging social construct as an added measure of a candidate's overall personality […]. Most PDs operate on the belief that the image an applicant portrays on an SN profile is a direct reflection of their qualities as a physician.[39]
4.1.5. Dealing with the impact of self-disclosure on the patient/doctor relationship (44)	[...] may risk a variety of repercussions if patients view this information, including a loss of trust or respect if patients believe depictions show irresponsible conduct on the part of the physician, potential conflict or disagreement if they learn that their physician holds religious or political views opposed to their own, or uncover other personal information about their physician that they find offensive.[40]
4.2. Challenge: Preventing inadequate stress relief through social media by posting patient sensitive information (15)	
	But the online banter between a group of doctors has provided a revealing glimpse of the secret terms they use to refer to patients and colleagues. In doing so they have started an online row between medics who think that their slang terms are offensive and unprofessional, and those who insist that black humor is an essential coping mechanism for people with highly stressful jobs"[41]
**5. Commitment to improving quality of care**	
5.1. Opportunity: Care improvement via advanced information sharing and multidisciplinary approach (27)	
	Clinical records can be gathered by one clinician for a case that requires multidisciplinary treatment, so that all the clinicians potentially involved can access the material and give their input towards the treatment plan and its completion.[14]
5.2. Opportunity: Care improvement via online feedback to providers (19)	
	Reading what patients are tweeting about your organization may help you better recognize their needs and concerns. More importantly, it also gives you an easy way to respond and let those patients be heard.[42]
5.3. Opportunity: Reducing power imbalances through social media (8)	

	If the patient feels intimidated or does not want to appear stupid in the physicians' eyes, it can be difficult for the patient to speak his or her mind or to ask questions concerning his or her health. In many ways social networking can help reduce this distance by empowering and educating patients.[…] Reducing power distances to patients have shown to improve patient confidence in starting, stopping, or making changes to treatment regimens.[43]
**6. Commitment to improving access to care**	
6.1. Opportunity: Facilitating online medical assistance through social media (28)	
	Physicians can use or create social media tools to assist patients who are adopting healthier behaviors or lifestyles, such as Facebook groups or Twitter sites to assist patients who have recently quit smoking.[31]
6.2. Challenge: Addressing concerns about medical information quality on social media platforms (27)	
	According to a recent study, 42% of adults report that they or someone they know gets medical advice online. Although the potential benefit of widely disseminated information and communication is ideal under many circumstances, opportunities for misrepresentation and the distribution of misleading information persist. For example, in 2008, there were 1,434 medical-related blogs; however only 279 were actually written by medical professionals.[44]
6.3. Challenge: Managing access to Internet technology in order to engage in social media (7)	
	Information is accessible to everyone, at any time. The rate limiting step to this empowerment is access to the technology however.[43]
**7. Commitment to a just distribution of finite resources**	
**8. Commitment to scientific knowledge**	
8.1. Opportunity: Supporting the access to scientific media via online links (9)	
	The results of this study suggest that social media sites such as YouTube are characterized by a large proportion of content provided by laypersons. However, there seems to be trend toward an increasing number of journals and academic institutions making use of this medium with their own YouTube channels for educational purposes.[45]
**9. Commitment to maintaining trust by managing conflicts of interest**	
9.1. Challenge: Maintaining transparency and dealing with advertisement and lobbying on social media platforms (3)	
	As many as 29% of health care–related social media authors report being approached by advertisers to endorse certain products. […] Currently, the FTC holds site administrators responsible for voluntarily and clearly identifying any financial relationship to a product or service they endorse on any social networking website, further reinforcing the need for transparency within health care–related social networking websites.[44]
**10. Commitment to professional responsibilities**	
10.1. Challenge: Allowing new dimensions of physicians' self-regulation through the usage of social media (104)	
	As members of a self-regulating profession, physicians who observe unprofessional content that has been posted by colleagues have an ethical obligation to address the situation. Ultimately, this responsibility derives from physicians' professional commitment to protect the welfare and trust of the public […][40]
10.2. Challenge: Adequate disciplinary actions due to breaches of professionalism on social media platforms (36)	
	Universities have disciplined professional students regarding the content of social media postings, with punishment ranging from informal warnings to dismissals. Also, the development and revision of university guidelines regarding professionalism and social media has increased.[46]
10.3. Challenge: Ensuring public trust while engaging on social media platforms (108)	
	Physicians certainly have the right to have private lives and relationships in which they can express themselves freely, but they must also be mindful that their patients and the public see them first and foremost as professionals, rather than as private individuals, and view physicians' conduct through the lens of their expectations about how an esteemed member of the community should behave. Thus, physicians must weigh the potential harms that may arise from presenting anything other than a professional presence on the Internet against the benefits of social interactions online.[40]
10.4. Challenge: Maintaining appropriate student/teacher relationship when interacting online (11)	
	The majority of responding clerkship directors believed that it was inappropriate to initiate or to accept a friend request from a current student but felt that this was more acceptable if he or she was a former student, that is, a student with whom the faculty member no longer had an evaluative role or who had graduated from the institution.[47]
